# Association Between a Refined Multidisciplinary Team-Based Care Model and Scalp Cooling Efficacy for Chemotherapy-Induced Alopecia Outcomes Among Breast Cancer Patients: A Single-Institution Retrospective Before–After Study

**DOI:** 10.3390/healthcare14142148

**Published:** 2026-07-16

**Authors:** Nobuko Tamura, Yukiko Nagaoka, Yukari Sano, Yoko Kobayashi, Kiyo Tanaka, Michiko Kurikawa, Akio Shibata, Yuko Tanabe, Takeshi Yamaguchi, Tomoko Hiraka, Ayumi Abe, Akie Kanamori, Yuko Kakimoto, Hideko Shimizu, Mikako Osaka, Yasuhiro Oda, Mayumi Ogura, Ryusuke Okamoto, Hidetaka Kawabata

**Affiliations:** 1 Department of Breast and Endocrine Surgery, Toranomon Hospital, Tokyo 105-8470, Japan; 2 Okinaka Memorial Institute for Medical Research, Tokyo 105-8470, Japan; 3 Cancer Chemotherapy Nursing, Toranomon Hospital, Tokyo 105-8470, Japan; 4 In-Hospital Beauty Salon, ANKS, Toranomon Hospital, Tokyo 105-8470, Japan; 5 Department of Medical Oncology, Toranomon Hospital, Tokyo 105-8470, Japan; 6 Department of Pharmacy, Toranomon Hospital, Tokyo 105-8470, Japan; 7 Department of Pathology, Toranomon Hospital, Tokyo 105-8470, Japan

**Keywords:** breast cancer, chemotherapy-induced alopecia (CIA), scalp cooling therapy (SCT), multidisciplinary framework

## Abstract

**Background:** Chemotherapy-induced alopecia (CIA) significantly impacts patient quality of life (QoL). Scalp cooling therapy (SCT) is effective, but its success in clinical settings remains variable. **Objective:** To evaluate the association between a refined multidisciplinary team-based approach, integrating appearance care professionals, and SCT efficacy in breast cancer patients. **Methods:** This retrospective before–after single-institution study compared SCT outcomes before (Group A, *n* = 66) and after (Group B, *n* = 79) implementing a refined care model among patients who completed their full planned chemotherapy courses. The baseline characteristics were comparable except for a significantly higher proportion of patients starting a weekly regimen in Group B (54.4% vs. 30.3%, *p* = 0.004). The model featured enhanced staff training, a specialized hair/scalp charting system, and task-sharing between nurses and integrated certified hairdressers. The primary endpoint was the number of cycles with Grade 2 or higher alopecia (CTCAE v5.0). **Results:** The refined approach was associated with improved scalp cooling outcomes. The percentage of patients with Grade 2+ alopecia at treatment completion decreased from 53.0% in Group A to 32.9% in Group B (*p* = 0.015). The overall proportion of time spent with Grade 2+ alopecia fell from 17.3% to 6.5% (*p* < 0.001). In the ddAC f/b wPTX regimen, the median number of Grade 2+ cycles reduced from 10.0 to 4.0 (*p* = 0.033). While the wPTX f/b ddAC regimen showed a numerical reduction (1.0 vs. 0.0 cycles), it did not reach statistical significance (*p* = 0.326). **Conclusions:** The refined multidisciplinary approach, including the integration of appearance care professionals, was associated with improved scalp cooling outcomes and lower median cycle numbers across regimens. Although these findings may be partly influenced by the baseline imbalance in chemotherapy regimens, this comprehensive team-based care model offers a feasible strategy for enhancing patient support in oncology settings.

## 1. Introduction

### 1.1. Background on Chemotherapy-Induced Alopecia (CIA) and Scalp Cooling Therapy (SCT)

Chemotherapy-induced alopecia (CIA) represents one of the most distressing and psychologically impactful side effects of cancer treatment, significantly affecting patients’ quality of life (QoL) and body image. The emotional burden of hair loss can lead to anxiety, depression, social withdrawal, and in some cases, non-adherence to potentially life-saving chemotherapy regimens [1]. A Japanese study has quantitatively assessed these appearance changes, confirming their direct correlation with patient distress [2]. Moreover, a subset of patients experience permanent alopecia, highlighting the need for effective prevention strategies [3]. Scalp cooling therapy (SCT) has emerged as a proven method for preventing or mitigating CIA by inducing vasoconstriction in the scalp, thereby reducing blood flow and the amount of chemotherapy drugs reaching the hair follicles. Its effectiveness has been demonstrated across various chemotherapy regimens in major randomized controlled trials [4,5] and large-scale real-world cohort studies [6].

### 1.2. Challenges in SCT Implementation and the Imperative of High-Quality, Team-Based Care

Despite its proven benefits, the widespread and consistently effective implementation of SCT faces various challenges. These include variability in reported efficacy across institutions and between different chemotherapy regimens [6], and the need for meticulous, precise cooling techniques. In many facilities, particularly within the Asian context, nurses often bear the sole responsibility for SCT implementation, leading to considerable workload burden and potentially suboptimal alopecia prevention outcomes [7,8,9,10]. This situation highlights a critical gap, as isolated efforts by individual healthcare providers, no matter how dedicated, may struggle to achieve consistently high efficacy. Achieving optimal and consistent SCT outcomes necessitates not only the right technology but also a robust and well-coordinated care-delivery model. In modern cancer care, a comprehensive multidisciplinary team approach is increasingly recognized as fundamental for providing holistic patient support and optimizing clinical outcomes. This extends beyond medical aspects to encompass patient education, psychological support, and meticulous procedural execution. The Common Terminology Criteria for Adverse Events (CTCAE) version 5.0 provides a standardized system for classifying adverse events, including alopecia, in clinical trials [11].

### 1.3. The Evolving Role of Appearance Care Professionals in Integrated Oncology Teams

Beyond purely medical interventions, addressing patients’ psychosocial and appearance-related concerns is vital for enhancing QoL during cancer treatment. Appearance care professionals, such as hairdressers and cosmetologists, possess unique expertise in managing hair and scalp health and can provide invaluable practical guidance and emotional support to patients experiencing hair changes due to chemotherapy and scalp cooling. Recent clinical trials have begun to address the long-term challenge of persistent alopecia [5], underscoring the importance of specialized and sustained care. Their active involvement in the care pathway can ensure proper scalp cooling technique, advise on hair care during and after treatment, and offer personalized strategies for managing hair loss or regrowth. At our institution, the formal integration of certified hairdressers into the multidisciplinary oncology team significantly refined our scalp cooling program. Their key roles included providing precise, hands-on assistance with cap placement and fitting, which is crucial for optimal cooling contact. Furthermore, we implemented a specialized charting system solely for hair, scalp, and cranial morphology information, facilitating seamless and detailed information sharing among nurses and hairdressers. This comprehensive approach, including a clear task-sharing framework between nurses and hairdressers, optimized the overall efficiency and effectiveness of SCT delivery.

However, despite their clear potential, the direct impact of formally integrating these specialists into oncology multidisciplinary teams, particularly within SCT protocols, remains an area requiring further investigation.

### 1.4. Study Objective

Drawing upon our single-institution experience, this study aims to evaluate the impact of a refined team-based approach, encompassing enhanced staff training, optimized protocols, and the active involvement of appearance care professionals, on overall scalp cooling therapy efficacy in breast cancer patients. We hypothesize that a holistic improvement in care quality through multidisciplinary collaboration and specialized appearance support leads to significantly better outcomes in preventing chemotherapy-induced alopecia.

## 2. Methods

### 2.1. Study Design and Setting

This study employed a retrospective observational design, comparing two distinct cohorts of breast cancer patients who opted for scalp cooling (SC) during perioperative chemotherapy at our institution. Group A comprised 75 patients who initiated SC between August 2021 and October 2022, representing the initial phase of SC implementation. Of these, 9 patients were excluded from analysis: 4 did not complete chemotherapy, 2 discontinued scalp cooling while continuing chemotherapy due to scalp-cooling-related discomfort (including headache or cold intolerance), and 3 did not consent to hair loss evaluation. The remaining 66 patients were included in the final analysis. Group B consisted of 93 patients who started SC between October 2023 and December 2024, following the implementation of significant team-based care enhancements and refined protocol optimization. Of these, 14 patients were excluded: 4 did not complete chemotherapy, 4 discontinued scalp cooling while continuing chemotherapy due to scalp-cooling-related discomfort (including headache or cold intolerance), and 6 did not consent to hair loss evaluation. The remaining 79 patients were included in the final analysis. This study was approved by the Toranomon Hospital Institutional Review Board (IRB) (Approval No. 2420). The research was conducted in accordance with the ethical standards of the responsible committee on human experimentation (institutional and national) and with the Helsinki Declaration of 1975, as revised in 2013, and the Ethical Guidelines for Medical and Health Research Involving Human Subjects (established on 23 March 2021) in Japan.

### 2.2. Patient Cohorts

This study included patients who received chemotherapy regimens for breast cancer and underwent scalp cooling therapy (SCT) at our institution. To be eligible for inclusion, patients had to have a confirmed diagnosis of breast cancer, be scheduled for perioperative chemotherapy, and have successfully completed their full planned chemotherapy regimen concurrently with SCT until treatment completion. Patients were excluded from this analysis if they met any of the following criteria: (1) incomplete chemotherapy with SCT, (2) incomplete medical or longitudinal alopecia evaluation records, (3) prior exposure to chemotherapy or cranial radiation therapy, or (4) pre-existing history of severe alopecia or active scalp disorders prior to treatment initiation. Consequently, a total of 145 patients who met all strict completion and evaluation criteria were included in the final analysis: 66 in Group A and 79 in Group B.

Patient background characteristics, including age, clinical stage, hormonal receptor status, HER2 status, and chemotherapy setting (preoperative or postoperative), were retrospectively collected from medical records. The median age was 50 years (IQR: 43.5–58.5) for Group A and 47 years (IQR: 40–54) for Group B. The baseline patient characteristics for both groups are summarized in Table 1. While most patient background characteristics showed no statistically significant differences between Group A and Group B—including age (*p* = 0.36), clinical stage (*p* = 0.35), hormonal receptor status (*p* = 0.15), HER2 status (*p* = 0.35), and chemotherapy setting (*p* = 0.87)—a significant baseline imbalance was noted in the type of treatment delivery. Specifically, the proportion of patients starting a weekly regimen was significantly higher in Group B than in Group A (54.4% vs. 30.3%, *p* = 0.004). The potential confounding effects of this regimen imbalance on overall SCT efficacy are critically evaluated in Section 4.

### 2.3. Intervention: The Refined Team-Based Approach

The refined team-based approach implemented for Group B focused on three key, interconnected areas: enhanced staff training, optimized protocols, and the active integration of appearance care professionals.

To enhance staff proficiency and minimize procedure variability, oncology nurses and certified hairdressers conducted iterative, collaborative hands-on training sessions prior to the Group B implementation. Rather than following a rigid classroom curriculum, the team focused on practical, peer-to-peer simulated practice, working together to master appropriate cap sizing, precise cap-fitting techniques, and strict adherence to cooling timelines. This continuous collaborative practice served to standardize the procedure and build a shared understanding of cap alignment between the two professions before managing patients independently.

Most notably, a total of three certified hairdressers from the in-hospital beauty salon (ANKS), all holding the national hairdresser license (Biyoshi Menkyo) under Japanese law, were formally integrated into our multidisciplinary oncology team to provide specialized, hands-on support. Their crucial role included assisting with precise cap placement and fitting, which is essential for maximizing contact and optimal cooling. To facilitate seamless communication and comprehensive patient management, we developed and implemented a specialized charting system dedicated solely to hair, scalp, and cranial morphology information. This specialized chart tracked objective parameters, including head circumference, baseline hair density, scalp sensitivity, and specific cranial shape variations, to ensure a personalized fit for the cooling cap. This system allowed for detailed, real-time, and consistent information sharing between nurses and hairdressers at each chemotherapy cycle.

Furthermore, a clear framework for task-sharing between nurses and hairdressers was established to optimize the overall efficiency and effectiveness of SCT delivery. Under this operational protocol, oncology nurses maintained sole responsibility for medical assessments, vital signs monitoring, and chemotherapy administration. Meanwhile, the integrated certified hairdressers managed technical cap adjustments, evaluated hair preservation status, and monitored scalp comfort during the cooling process. Beyond these technical aspects, hairdressers also offered invaluable practical hair care advice during treatment and provided crucial psychosocial support to patients. These comprehensive enhancements collectively aimed to elevate the quality of care and ensure more consistent and effective scalp cooling outcomes.

### 2.4. Scalp Cooling Protocol

Scalp cooling was performed using the Paxman Cooling system, strictly adhering to the manufacturer’s recommended protocol. This standard protocol involved a pre-cooling period of 30 min before chemotherapy infusion, followed by continuous cooling during the infusion, and a post-cooling period of 90 min after infusion completion. While both Group A and Group B followed identical manufacturer-defined baseline cooling parameters to ensure protocol consistency, the technical execution workflow for Group B was significantly optimized to secure high reproducibility. In Group A, the protocol was executed solely by bedside nurses, which occasionally introduced technical variability in cap alignment due to competing clinical workloads.

In Group B, the implementation involved a highly structured, multi-step collaborative process. Following the physician’s initial consultation, a dedicated nurse conducted a detailed medical assessment. Crucially, to standardize the specialized intervention, we established a standalone, dedicated scalp-cooling charting system utilized exclusively by the appearance care team. This independent chart recorded precise individual cranial morphology, head circumference, and hair density for each patient. By reviewing this dedicated record at every treatment session, any participating certified resident hairdresser could replicate the exact personalized cap fitting required for that specific patient’s head shape. This refined approach ensured rigorous, gap-free cap contact and strict adherence to cooling timelines across all chemotherapy cycles, successfully eliminating the inter-operator variability observed during the initial implementation phase (Supplementary Figure S1).

### 2.5. Outcome Measures

The primary outcome measure was the efficacy of SCT, evaluated by the number of chemotherapy cycles with Grade 2 or higher alopecia according to the Common Terminology Criteria for Adverse Events (CTCAE) version 5.0. Grade 2 alopecia was defined as a noticeable loss of hair, requiring an intervention (e.g., wig, scarf).

To ensure standardized assessment, alopecia grading was systematically performed by attending oncology nurses at the beginning of each chemotherapy cycle. As this study employed a retrospective medical record review design, the outcome assessors were not blinded to the patient cohorts (Group A vs. Group B), which represents a potential assessment bias.

Secondary outcomes included the percentage of patients with Grade 2 or higher alopecia at the end of treatment. Additionally, to evaluate longitudinal outcomes, two cycle-based measures were defined as follows:

1. The median proportion of cycles with Grade 2+ alopecia per patient, calculated as:

Proportion of Cycles

$$=\frac{Number\;of\;cycles\;with\;Grade\;2+}{Total\;number\;of\;chemotherapy\;cycles\;received\;by\;the\;individual\;patient}$$

2. The overall proportion of time spent in Grade 2+ alopecia across the entire cohort, calculated by pooling all observation cycles within each group as the denominator:

Overall Proportion of Time

$$=\frac{Total\;pooled\;number\;of\;Grade\;2+\;in\;the\;group}{Total\;pooled\;number\;of\;treatment\;cycles\;administered\;in\;the\;group}$$

Alopecia grading was performed by attending oncology nurses at each cycle. Although the retrospective design meant that outcome assessors were not blinded to the treatment cohorts (introducing a potential risk of assessment bias), all clinical grading strictly adhered to the standardized, objective criteria of CTCAE v5.0 to minimize inter-rater variability. Regarding scalp cooling tolerability, among patients who completed chemotherapy, the scalp cooling completion rate was 92.0% (69/75) in Group A and 91.4% (85/93) in Group B. The reasons for discontinuation were scalp cooling-related discomfort, specifically headache or cold intolerance. The specific reason for each individual case could not be determined from the retrospective chart review. Notably, even among the patients who successfully completed the full course of scalp cooling, a substantial number experienced transient, mild-to-moderate discomfort, including headaches and cold intolerance, during the cooling sessions. However, these symptoms were manageable, and no serious adverse events attributable to scalp cooling were observed in either group.

### 2.6. Statistical Analysis

To control for potential confounding associated with baseline discrepancies in chemotherapy characteristics, a multivariable linear regression analysis was performed. The dependent variable was defined as the longitudinal duration of alopecia, quantified as the proportion of completed chemotherapy cycles presented with Grade 2 or higher alopecia per patient. The forced-entry independent variables included the cohort group (Group A vs. Group B), the chemotherapy scheduling sequence (starting with a weekly regimen, coded as 0 [Yes, reference category] vs. 1 [No]), the inclusion of specific high-risk cytotoxic agents, (use of Anthracycline or Docetaxel/Cyclophosphamide [TC] regimens [Yes/No], recorded as “AC or TC use” in Table 3, see also Table 2 for the stratified analysis across regimens), and continuous baseline patient age. Unstandardized regression coefficients (*B*), standardized coefficients (β), and their respective 95% confidence intervals (CIs) were evaluated to determine independent associations. Because the primary dependent variable was a continuous proportion (the ratio of Grade 2 or higher cycles to total cycles), multivariable linear regression was applied after confirming that the model residuals satisfied normality assumptions. To account for variations in the total number of chemotherapy cycles completed by individual patients, the analysis was adjusted for the specific planned regimen sequence as a covariate. As a sensitivity analysis to address the bounded nature of the proportional outcome (0–1), a multivariable binary logistic regression was additionally performed using the presence or absence of Grade 2 or higher alopecia at treatment completion as a binary dependent variable (yes/no). The same independent variables were included, with the exception of the AC/TC regimen variable, which was excluded due to complete separation. The reference category for the cohort group was set as Group B.

## 3. Results

### 3.1. Patient Characteristics

A total of 145 breast cancer patients undergoing chemotherapy with scalp cooling were included in this study, comprising 66 patients in Group A (pre-intervention cohort) and 79 patients in Group B (post-intervention cohort) (Supplementary Figure S2). The baseline patient characteristics for both groups are summarized in Table 1.

The median age was 50 years (IQR: 43.5–58.5) in Group A and 47 years (IQR: 40–54) in Group B. No statistically significant differences were detected between Group A and Group B regarding age (*p* = 0.36), clinical stage (*p* = 0.35), hormonal receptor status (*p* = 0.15), HER2 status (*p* = 0.35), or chemotherapy setting (preoperative vs. postoperative, *p* = 0.87). However, these non-significant *p*-values should be interpreted with caution and do not definitively prove equivalence between the cohorts due to the retrospective study design. Notably, a major and statistically significant imbalance was observed in the treatment profiles: the proportion of patients starting a weekly regimen was substantially higher in Group B than in Group A (54.4% vs. 30.3%, *p* = 0.004). Given that weekly chemotherapy regimens are clinically associated with different baseline alopecia risks compared to multi-week regimens, this baseline discrepancy represents a critical confounding factor. This distinction will be further considered in the discussion regarding its potential implications for overall efficacy.

### 3.2. Overall Efficacy Improvement by Group (Intervention Effect)

The implementation of the refined team-based approach was associated with improvements in scalp cooling efficacy across the observed cohorts. As shown in Table 1, the percentage of patients with Grade 2 or higher alopecia at the end of treatment decreased from 53.0% in Group A to 32.9% in Group B (*p* = 0.015, unadjusted Risk Difference: −20.1%, 95% Confidence Interval [CI]: −35.8% to −4.4%). Furthermore, the overall proportion of time spent in Grade 2 or higher alopecia during the entire treatment period showed a reduction from 17.3% in Group A to 6.5% in Group B (*p* < 0.001). However, as noted in the baseline characteristics, these unadjusted overall comparisons must be interpreted with extreme caution, as they may be partially confounded by the higher proportion of weekly regimens in Group B.

To evaluate the intervention effect while minimizing the confounding impact of treatment regimens, stratified analyses were conducted for specific chemotherapy sequences (Table 2). Patients receiving the ddAC f/b wPTX regimen experienced improved outcomes in Group B, with the median number of cycles with Grade 2 or higher alopecia significantly reduced from 10.0 cycles (IQR: 7.0–12.0) in Group A to 4.0 cycles (IQR: 3.0–9.5) in Group B (*p* = 0.033). In contrast, for regimens with lower baseline alopecia risks or smaller sample sizes, the differences did not reach statistical significance. For the wPTX f/b ddAC regimen, patients showed a numerical reduction in the median number of Grade 2 or higher cycles from 1.0 (IQR: 0.0–4.0) in Group A to 0.0 (IQR: 0.0–2.0) in Group B (*p* = 0.326). Similarly, for the TC regimen, the median number of cycles with Grade 2 or higher alopecia was 1.0 (IQR: 0.0–2.0) in Group A and 0.0 (IQR: 0.0–1.0) in Group B (*p* = 0.326). These small subgroup findings are underpowered and should not be overinterpreted.

Nevertheless, the stratified results—particularly the significant reduction within the identical ddAC f/b wPTX sequence—suggest that standardized cap fitting and multi-professional coordination potentially contributed to stabilizing SCT execution, leading to more consistent alopecia prevention outcomes across different clinical settings.

Crucially, the multivariable linear regression analysis demonstrated that the protective clinical benefit of the refined care model remained highly robust even after implementing stringent statistical controls for complex chemotherapy baseline imbalances (Table 3). After simultaneously adjusting for the initiating cycle sequence, anthracycline or TC regimen exposure, and patient age, the implementation of the standardized multi-professional framework (Group B) was independently and significantly associated with a 12.5% reduction in the overall duration spent with Grade 2 or higher alopecia compared to the initial phase (Group A) (Unstandardized *B* = −0.125, Standardized β = −0.226, 95% CI: −0.204 to −0.046, *p* = 0.002). As clinically expected, the initiating chemotherapy sequence significantly influenced the longitudinal duration of alopecia. With the weekly start regimen as the reference category, patients who started with a multi-week dense-dose regimen (e.g., ddAC) were associated with a 26.0% higher proportion of cycles spent with Grade 2 or higher alopecia (Unstandardized *B* = 0.260, Standardized β = 0.465, 95% CI: 0.181 to 0.340, *p* < 0.001), consistent with the clinically higher alopecia burden associated with dense-dose chemotherapy schedules. In contrast, the net addition of anthracycline or TC status itself did not show an isolated independent correlation with longitudinal outcomes once the structural sequence timing was adjusted (Unstandardized *B* = 0.018, *p* = 0.431). These statistical findings clarify that the superior outcomes tracked in Group B were fundamentally driven by the structural precision of the team-based care model rather than an artifact of baseline patient allocation shifts. In a sensitivity analysis using multivariable binary logistic regression with Grade 2 or higher alopecia at treatment completion as the binary outcome, the findings were consistent with the primary analysis. With Group B as the reference category, Group A (pre-intervention cohort) was independently associated with significantly higher odds of Grade 2 or higher alopecia at treatment completion after adjusting for the initiating regimen sequence and patient age (OR = 2.292, 95% CI: 1.142–4.603, *p* = 0.020), supporting the robustness of the primary linear regression findings.

### 3.3. Methodological Context of the Multidisciplinary Framework

As described in Section 2, the refined care model implemented in Group B was evaluated as a comprehensive, multifaceted intervention package; therefore, specific quantitative data isolating the independent contribution of appearance care professionals (such as certified hairdressers) was not separately measured via numerical efficacy metrics.

The active involvement of these specialists served as an operational component within the broader multidisciplinary approach. Their presence was structured to support the overall execution of scalp cooling by facilitating personalized cap application and providing standardized practical guidance on hair care during and after treatment. Consequently, the observed improvements in Group B reflect the combined clinical effect of staff training, standardized charting, and multi-professional coordination, rather than a single isolated factor. The hypothesized qualitative mechanisms and clinical implications of integrating appearance care specialists into the oncology team will be further addressed in Section 4.

## 4. Discussion

### 4.1. Summary of Key Findings

This retrospective observational study evaluated the clinical outcomes of a refined, multidisciplinary team-based care model designed to optimize scalp cooling therapy (SCT) delivery for breast cancer patients. Following the sequential implementation of enhanced staff training, standardized charting, and the integration of certified hairdressers, we observed a significant reduction in the percentage of patients presenting with Grade 2 or higher alopecia at treatment completion, as well as a lower overall proportion of time spent with significant hair loss compared to the pre-intervention cohort (Group A).

While these unadjusted findings must be interpreted cautiously due to baseline imbalances in chemotherapy regimens, the stratified analysis within the identical ddAC f/b wPTX sequence revealed a notable reduction in the median number of Grade 2+ cycles. These results suggest that a structured, multi-professional execution model is strongly associated with improved real-world SCT outcomes and possesses the potential to enhance supportive care systems aimed at mitigating both acute distressing hair loss and the long-term risk of persistent alopecia [1].

### 4.2. Interpretation of Findings in the Context of the Existing Literature

Our findings align with and expand upon the foundational literature emphasizing the importance of a multidisciplinary approach in supportive cancer care and the critical role of quality improvement initiatives. The baseline efficacy of SCT has been well established by landmark randomized controlled trials [3,4] and large-scale real-world registry data [5], which generally report hair preservation rates ranging from 50% to 65% depending on the chemotherapy regimen. Our findings are particularly noteworthy in the context of previous studies suggesting potentially lower SCT efficacy in Asian patients [9]. Several reports have indicated that scalp cooling may be less effective in Asian populations compared to their Caucasian counterparts, a phenomenon potentially influenced by structural differences in hair characteristics, such as higher hair thickness, lower follicle density, and distinct hair shaft morphology [12]. While these biological barriers often complicate cap-to-scalp contact, our stratified results—particularly within the identical ddAC f/b wPTX sequence—suggest that a refined, team-based approach incorporating technical application adjustments has the potential to mitigate some of these institutional disparities, making successful SCT execution less dependent on patient ethnicity and more on the precision of the care delivery model.

However, a rigorous interpretation of our overall outcomes requires careful consideration of the significant baseline regimen imbalance. Group B included a substantially higher proportion of patients receiving a weekly regimen (54.4% vs. 30.3%), which clinically carries a lower baseline risk of severe Grade 2+ alopecia compared to dense-dose multi-week anthracycline regimens. Since chemotherapy regimen and sequence are primary determinants of hair loss and cooling success, this unadjusted imbalance represents a major limitation, and the observed overall improvements in Group B may be partly driven by this treatment profile discrepancy rather than the care model alone.

Furthermore, the active integration of certified hairdressers must be interpreted as one contributing component within a comprehensive multidisciplinary framework, rather than an independently proven causal factor. As a part of this integrated care model, appearance care professionals likely supported clinical outcomes through multiple combined avenues:

*Technical Support*: Utilizing specialized knowledge of hair anatomy and cranial contours to minimize air gaps during cap placement, which is vital for optimal cooling conduction.
*Adherence and Education*: Enhancing patient comfort and providing practical, real-world hair care advice to empower patients throughout the treatment period.
*Psychosocial Care*: Addressing appearance-related anxieties directly at the point of care, which complements the medical team’s role and supports overall well-being.

Recent landmark findings on the importance of preventing even persistent chemotherapy-induced alopecia further underscore the clinical value of optimization efforts in supportive care [13]. By framing appearance care as a structured multi-professional process, oncology teams can better address the nuanced, multi-dimensional challenges of real-world SCT implementation.

### 4.3. Clinical Implications

The results of this study offer practical insights for oncology institutions seeking to enhance the quality of their SCT delivery systems. Our findings highlight that maximizing the real-world efficacy of scalp cooling extends beyond the acquisition of cooling technology; it is highly associated with a commitment to continuous quality improvement, collaborative staff preparation, and a structured multi-professional execution model.

While our specific setting benefited from a dedicated, in-hospital beauty salon structure, this experience suggests that integrating appearance care expertise can play a supportive role in refining both technical cap adjustments and psychosocial patient care. Rather than implying a one-size-fits-all model immediately generalizable to all clinical environments, these insights advocate for institutional flexibility. Oncology teams can explore feasible methods to bridge the implementation gap, whether through formal specialist integration or by adapting multi-professional checklists to reduce inter-operator variability and improve the overall patient care experience.

### 4.4. Limitations

This study has several major limitations inherent to its retrospective, single-institution design. First, causality cannot be definitively established due to its observational nature, and unmeasured confounding variables may exist. Second, although a significant baseline imbalance was present in the chemotherapy regimens between the two groups (with Group B including a substantially higher proportion of weekly regimen; *p* = 0.004), we implemented a robust multivariable linear regression analysis to statistically control for these specific treatment profile discrepancies (Table 3). Nevertheless, because this was a retrospective single-institution study, residual unmeasured confounding cannot be entirely ruled out. Third, because this was a retrospective chart review, the oncology nurses who performed the CTCAE alopecia grading at each cycle were not blinded to the patient cohorts, which introduces a potential risk of assessment bias. Fourth, the multi-year cohort design spanning from 2021 to 2024 introduces potential temporal bias; the clinical team naturally developed a learning curve and institutional familiarity with the Paxman system over time, which could independently enhance cooling outcomes regardless of the refined model. While the refined team-based approach was multifaceted, it is challenging to isolate the precise independent contribution of each individual component (e.g., staff training, standardized charting, or certified hairdresser involvement) to the overall improvement. Finally, although the manuscript discusses the clinical importance of patient comfort, anxiety relief, and quality of life (QoL), patient-reported outcomes (PROs), satisfaction scores, and distress measures were not quantitatively collected in this retrospective registry, limiting our ability to verify these hypothesized psychosocial benefits. Furthermore, while our high completion rates (>91%) demonstrate clinical feasibility, it is clinically important to note that completion did not mean symptom-free; rather, most completing patients tolerated varying degrees of scalp discomfort, headache, or cold intolerance throughout their cycles, successfully managing to continue the therapy with institutional support. Additionally, patients who discontinued scalp cooling (2 in Group A and 4 in Group B) were excluded from the primary analysis, as longitudinal alopecia grading data were unavailable for these individuals. A formal sensitivity analysis including these patients was not feasible due to the absence of complete outcome data. This exclusion may have introduced a selection bias toward patients with higher tolerability, potentially overestimating the scalp-cooling completion and efficacy rates reported in this study. Furthermore, this study primarily focused on overall efficacy improvements across regimens; a more detailed analysis of the impact of specific chemotherapy regimen sequencing on SCT outcomes, which also contributed to protocol optimization, will be presented in a separate publication.

### 4.5. Future Directions

Future research should utilize prospective, multicenter studies to validate these preliminary findings across broader, ethnically diverse patient populations and varied institutional settings. To address the limitations of the current retrospective analysis, subsequent trials should incorporate rigorous multivariable regression adjustments or propensity score matching to fully control for baseline chemotherapy regimen and sequence imbalances.

Further investigation into the specific mechanisms by which appearance care professionals influence SCT efficacy remains highly valuable. Specifically, future protocols should systematically collect validated patient-reported outcomes (PROs)—including standardized quality of life (QoL) questionnaires, body image scales, patient satisfaction scores, and psychological distress measures—to quantitatively evaluate the hypothesized psychosocial benefits of integrated appearance care. Additionally, qualitative research exploring patient experiences and mixed-methods compliance tracking would clarify behavioral adherence pathways. Finally, studies exploring the cost-effectiveness of integrating certified appearance care specialists into routine oncology care are warranted to provide the economic evidence necessary to support wider institutional adoption of this comprehensive multidisciplinary approach.

## 5. Conclusions

This study suggests that achieving optimal scalp cooling therapy (SCT) efficacy extends beyond technical execution alone; it is highly associated with the implementation of a comprehensive, high-quality, and multidisciplinary care model. Our findings highlight that a refined team-based approach, which includes the integration of certified appearance care professionals such as hairdressers, was associated with improved alopecia prevention outcomes in breast cancer patients undergoing chemotherapy. Although these unadjusted retrospective findings may be partially influenced by baseline imbalances in chemotherapy regimens, the active collaboration between oncology nurses and appearance care specialists has the potential to enhance both the practical application of SCT and the supportive care provided to patients. Ultimately, this multi-professional coordination offers a feasible and promising institutional framework for optimizing real-world SCT delivery and enhancing comprehensive patient support in oncology practice.

## Figures and Tables

**Table 1:** Patient background characteristics in Group A and Group B.

		Group A (*n* = 66)	Group B (*n* = 79)	*p* Value
Age	Median (IQR)	50 (43.5–58.5)	47 (40–54)	0.36
Stage	IA	22 (33.3%)	16 (20.3%)	0.35
	IIA	18 (27.3%)	25 (31.6%)	
	IIB	11 (16.7%)	10 (12.7%)	
	IIIA	9 (13.6%)	14 (17.7%)	
	IIIC	5 (7.6%)	10 (12.7%)	
	Local recurrence	1 (1.5%)	4 (5.1%)	
Subtype	Luminal	26 (39.4%)	41 (51.9%)	0.383
	Luminal HER2	16 (24.2%)	18 (22.8%)	
	Pure HER2	8 (12.1%)	5 (6.3%)	
	Triple negative	16 (24.2%)	15 (19.0%)	
Hormonal receptor	Positive	42 (63.6%)	59 (74.7%)	0.15
	Negative	24 (36.4%)	20 (25.3%)	
HER2	Positive	24 (36.4%)	23 (29.1%)	0.35
	Negative	42 (63.6%)	56 (70.9%)	
Chemotherapy	Preoperative	30 (45.5%)	37 (46.8%)	0.87
	Postoperative	36 (54.5%)	42 (53.2%)	
Regimen	ddAC → wPTX *	23 (34.8%)	15 (19.0%)	
	ddAC → DTX *	3 (4.5%)	1 (1.3%)	
	wPTX * → ddAC	14 (21.2%)	27 (34.2%)	
	DTX * → ddAC	2 ((3.0%)	0(0.0%)	
	PEM/Cb/wPTX → PEM/AC	1 (1.5%)	12 (15.2%)	
	TC *	17 (25.8%)	20 (25.3%)	
	Weekly PTX *	6 (9.1%)	4 (6.9%)	
Starting a weekly regimen	Yes	20 (30.3%)	43 (54.4%)	0.004
	No	46 (69.7%)	36 (45.6%)	
Patients with Grade 2+ Alopecia at End of Treatment (%)	Yes	35 (53.0%)	26 (32.9%)	0.015
	No	31 (47.0%)	53 (67.1%)	
Median Proportion of cycles with Grade 2 or higher alopecia		0.33	0.15	<0.01
Overall Proportion of Time in Grade 2+ Alopecia (%)		17.3%	6.5%	<0.001

* if HER2 positive, anti HER2 drugs added taxane.

**Table 2:** Median number of cycles with G2 or higher alopecia.

Median (IQR)	*n*	Group A	Group B	*p* Value
All	145	2.0 (0.0–8.0)	0.0 (0.0–2.0)	0.001
wPTX * → ddAC	41	1.0 (0.0–4.0)	0.0 (0.0–2.0)	0.326
ddAC → wPTX *	38	10.0 (7.0–12.0)	4.0 (3.0–9.5)	0.033
TC *	37	1.0 (0.0–3.0)	0.0 (0.0–2.0)	0.326

* if HER2 positive, anti HER2 drugs added taxane.

**Table 3:** Multivariable linear regression analysis for the duration of Grade 2+ alopecia.

Variable	Unstandardized Coefficient *B*	Standardized Coefficient β	95% Confidence Interval (CI)	*p* Value
Cohort Group (Group A vs. Group B)	−0.125	−0.226	−0.204 to −0.046	0.002
Weekly start Regimen (No vs. Yes as reference)	0.260	0.465	0.181 to 0.340	<0.001
AC or TC Use (Yes vs. No)	0.018	0.055	−0.026 to 0.061	0.431
Age (per year)	0.001	0.022	−0.003 to 0.004	0.760

Note: The model’s overall fit was stable with an R2 of 0.321, Adjusted R2 = 0.301, F = 16.520, *p* < 0.001. For the cohort group variable, Group A is treated as the reference category. For the weekly start regimen variable, the weekly regimen (Yes) is treated as the reference category (coded as 0).

## Data Availability

The data presented in this study are available on reasonable request from the corresponding author. The data are not publicly available due to institutional restrictions and patient privacy regulations.

## Supplementary Materials

The following supporting information can be downloaded at: https://www.mdpi.com/article/10.3390/healthcare14142148/s1, Figure S1: Scalp Cooling Therapy: Cosmetologist Role—Group A vs. Group B; Figure S2: Study Population Flow: Group A (Control) vs. Group B (Intervention).
