# Supplementary material for: Association Between a Refined Multidisciplinary Team-Based Care Model and Scalp Cooling Efficacy for Chemotherapy-Induced Alopecia Outcomes Among Breast Cancer Patients: A Single-Institution Retrospective Before–After Study
Source: Healthcare (Basel). 2026 Jul 16;14(14):2148. doi: 10.3390/healthcare14142148 (PMC13411545; doi:10.3390/healthcare14142148)

Figure S1. Scalp Cooling Therapy: Cosmetologist Role — Group A vs. Group B

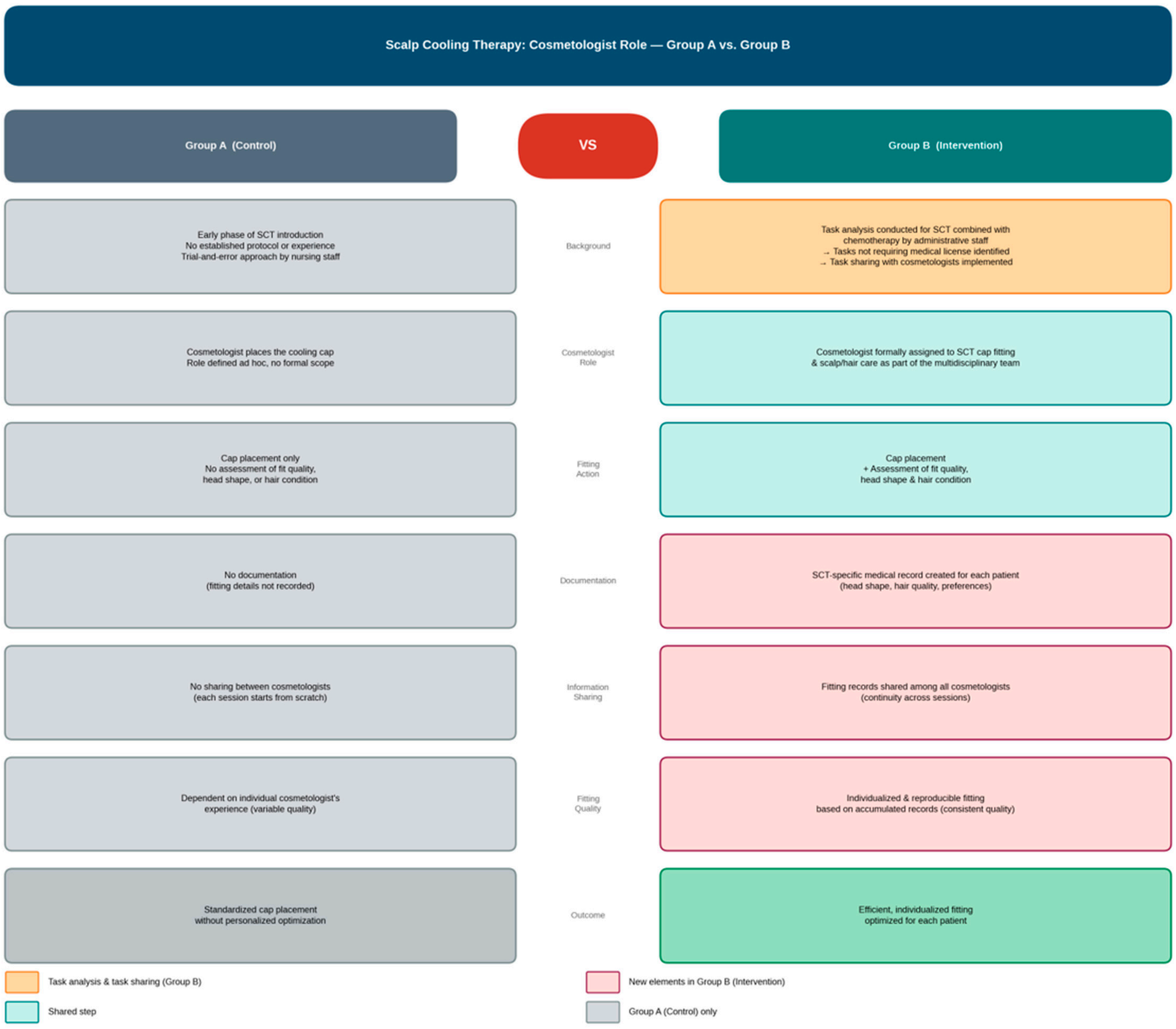

Figure S2. Study Population Flow: Group A (Control) vs. Group B (Intervention)

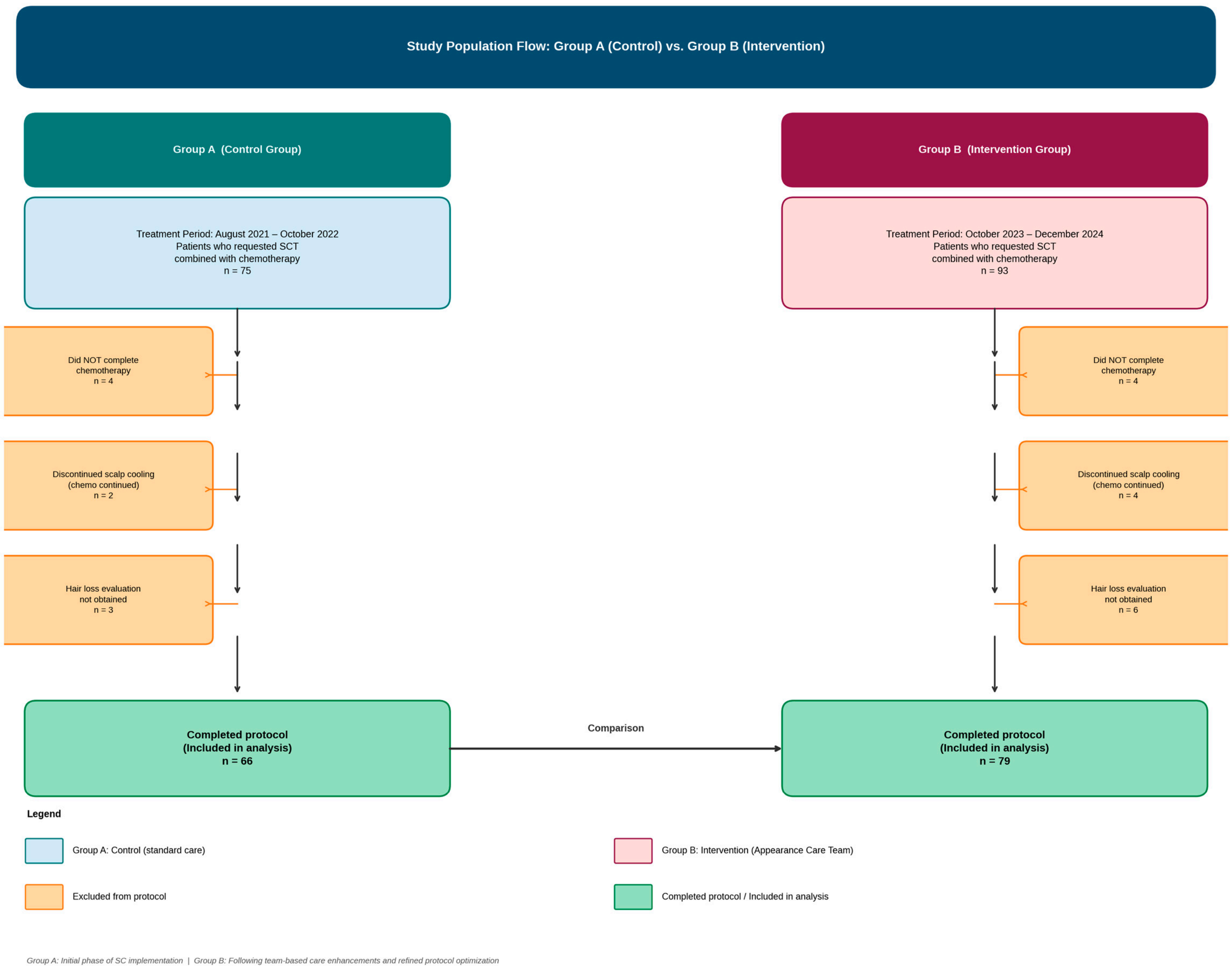

Supplement: Supplementary file 1 [file healthcare-14-02148-s001.zip › healthcare-4379891-supplementary.pdf]
